# Short report: autistic gastrointestinal and eating symptoms treated with secretin: a subtype of autism

**DOI:** 10.1186/1745-0179-1-24

**Published:** 2005-11-15

**Authors:** Stefano Pallanti, Stefano Lassi, Giampaolo La Malfa, Marco Campigli, Roberto Di Rubbo, Giulia Paolini, Valentina Cesarali

**Affiliations:** 1 Department of Psychiatry, University of Florence, Italy; 2 Institute of Neuroscience, Florence, Italy; 3 SIRM (Italian Society for the study of Mental Retardation), Via Gordigiani, 58, 50127, Firenze, Italy

## Abstract

Pervasive Developmental Disorders (PDD) are chronic, lifelong disorders for which there is as yet no effective cure, and medical management remains a challenge for clinicians. The current report describes two patients affected by autistic disorder with associated gastrointestinal symptoms.

They received multiple doses of intravenous secretin for a six-month period and were assessed with several specific outcome measures to evaluate drug effect.

The administration of secretin led to some significant and lasting improvement in only one case.

Gastroesophageal reflux may contribute to some of the behavioural problems and explain the effect of secretin since its suppressive effect on gastric secretion is well known. It is also true that autistic children with gastroesophageal reflux and a higher IQ could constitute a subtype which responds to secretin administration and that could be labelled as a "gastrointestinal subtype".

## Introduction

Pervasive Developmental Disorders (PDD) are chronic, lifelong disorders for which there is as yet no effective cure, and medical management remains a challenge for clinicians. In spite of improvements in some associated "problematic behaviors" with specific drugs, effective medical treatment for the core language- and social cognition-related symptoms are not available because the biology is not clearly understood and thus proper drug treatment has not been possible [1]. However, significant advances are being made towards understanding the mechanisms of the disorders, and major challenges lie ahead in evaluating the growing number of treatments for autism and in integrating the results of research into treatment and educational settings [2].

Since the experience of Horvath *et al*. [3] regarding secretin administration, with their report of "a dramatic improvement in the behavior of autistic children, manifested by improved eye contact, alertness, expansion of expressive language", and "relief of gastrointestinal symptoms", particular attention has been given to the potential role of this biological agent on autism.

Interest in secretin is also justified by many studies that have not only demonstrated the role of secretin as a classic hormone in the gastrointestinal system, but have firmly supported its neuropeptide role [4], since secretin has been shown to be capable of crossing the blood-brain barrier [5] and of depolarizing nucleus tractus solitarius neurons [6], activating brain regions including areas abnormal in autism [7].

Although preliminary reports [8] on secretin application initially generated enthusiasm, especially among parents of children with PDD [9], recent controlled studies seem to have dampened such enthusiasm (for a review, see [10]), although there is still some space for discussion.

A study by Sandler *et al*. [11] showed the lack of benefits in the treatment of core autistic symptoms with a single dose of secretin, though Sandler recognized that his study had several limitations: first, the follow-up period was short-term; second, only a single dose was administered; third, the diagnostic schedules used were not specific enough to measure the response to treatment.

Owley *et al*. [12] also conducted a double-blind versus placebo-controlled trial of porcine secretin for the treatment of autism, reaching the conclusion that there is no evidence of its efficacy.

Various studies conducted between 2000 and 2002 to assess the efficacy of a single dose of secretin on autistic features reported no significant effects [13]. All these studies adopted the DSM-IV criteria [14] and rating scales, which mainly focus on the so-called core autistic symptoms (only Corbett *et al*., [13] also used gastrointestinal measures to evaluate drug effects).

The wide range of autistic symptoms and their correlation with gastrointestinal functions, eating behavior and social interaction have not been focused on since Lightdale *et al*. [15] reported, following a single-blind, open-label pilot study, no effect of secretin in a five-week period on the language and behavior of 20 children with autistic and gastrointestinal symptoms.

Unis *et al*. [16] in a randomized double-blind, placebo-controlled study, reported no evidence that either biologic or synthetic secretin provided amelioration of symptoms beyond placebo. Likewise, Levy *et al*. [17] came to the conclusion that a single dose of secretin is not effective in changing behavior and communication in children with PDD if compared to placebo. In a controlled setting [18], parents of children with autism treated with a single dose of secretin were unable to distinguish the short behavioral effects of secretin from placebo.

These recent controlled studies seemed to spell the death knell for an unproven treatment that captured the public's imagination but found support in only a few positive reports [19].

However, there are several limitations in these very appreciable papers that leave some scope for us to report our observations. First, most of the studies were performed with a single infusion before the follow-up observational period. There are only three studies conducted on small samples [20] that report no evidence for the efficacy of repeated doses of secretin on the symptoms, language or cognitive functioning of children with autism. Second, the (paradoxically) large size of the sample when studying "categorized" autistic subjects leads to the risk of obtaining ungeneralizable data because the category of autism has a significant internal heterogeneity. Third, the wide range of typologies and the degrees of severity of symptoms have only been distinguished in with or without a widely defined condition labeled as "gastrointestinal symptoms", and conclusions cannot be drawn about hypothetically specific subtypes.

## Methods

Given the controversial views surrounding the utility of this hormone in the treatment of autism, we decided to report our experience and considerations regarding two children with a diagnosis of autistic disorder according to DSM IV criteria. They were treated with secretin in order to assess any kind of clinical improvement; particular attention was devoted to the complexity of autistic eating behavior.

### Participants

The first subject was a 9-year-old male, with a body weight of 35 kg, who was diagnosed as autistic at the age of two. We were able to confirm the previous diagnosis, as language and communication, social interaction and behavioral core symptoms were present; associated problems included a diet restriction and reflux esophagitis, and he cried at inappropriate times. He had an IQ of 99.

The second subject was a 7-year-old male with a body weight of 25 kg, who received his first diagnosis of autism at about the age of two. His IQ was 86. We confirmed the previous diagnosis, as he presented core symptoms and associated problems, especially gastrointestinal symptoms in the form of chronic diarrhea. The two boys were not taking other psychotropic medications during the study.

These two subjects were recruited consecutively, in a period when the data reported in literature about the potential effectiveness of secretin were promising. No other case was subsequently recruited (Table 1).

**Table 1:** Participant details.

Case	Age (yr)	Weight (kg)	IQ SB-IS	Gastrointestinal symptoms	Core symptoms
**1**	9	35	99	Reflux esophagitis Strict diet	Severe abnormal relationships Severe abnormal verbal communication Inappropriate crying Rocking Repetitive movements Abnormal adaptation to change Rituals Sleep problems
**2**	7	25	86	Chronic diarrhoea	Severe abnormal relationships Severe abnormal verbal communication Routine Obsessive interests Repetitive movements Self-injurious behaviour Tantrums

### Procedure

The method consisted of a single-blind protocol. The Stanford-Binet Intelligence Scale was employed before treatment in order to evaluate intelligence based on standardized criteria [21].

After obtaining informed consent from the parents, we administered aJapanese biological secretin, extracted from the duodenum of pigs, each vial containing 50 CU of secretin diluted in 2 ml. After a dose test of 1 CU over 1 minute, approximately 15 minutes prior to the full dose, carried out in order to determine possible allergic reactions, we administered the full intravenous secretin dosage of 2 CU/kg body-weight, given over a 1-minute period in a volume of 0.2 ml/kg, for each injection. The aim of the study was to administer 6 consecutive injections of secretin, one every 4 weeks. Each administration was carried out by an expert with proven experience of allergic anaphylactic reactions and resuscitation methods, because of the possibility that repeated use might result in an allergic response. Assessment included: 1. the Behavioral Summarized Evaluation [22], a 20-item scale that seemed a valid clinical tool to assess behavioral modifications and the evolution of the symptoms of children with autistic disorder; 2. the Clinical Global Impression Scale [23], a 3-item scale (Severity of Illness, Global Improvement, Efficacy Index) used to assess treatment response in psychiatric patients. We used these to measure eight separate features associated with autism (response to social interaction, social inition, use of speech, types of repetitive behavior, behavior problems, activity level, sleep problems, and digestive problems) on a standardized, seven-point Likert scale; 7 indicated "extremely ill", 4 "moderately ill" and 1 "normal" as regards Severity; 7 indicated "very much worse", 4 "no change", and 1 "very much improved" as regards Global Improvement; the Efficacy Index was measured on a four-point scale from "none" to "outweighs therapeutic effect"; 3. the Childhood Autism Rating Scale [24] which is the most widely used standardized instrument specifically designed to aid in the diagnosis of autism in young children [25], which includes 15 items (Relationships with People, Imitation, Affect, Use of Body, Relation to Non-human Objects, Adaptation to Environmental Change, Visual Responsiveness, Auditory Responsiveness, Near Receptor Responsiveness, Anxiety Reaction, Verbal Communication, Nonverbal Communication, Activity Level, Intellectual Functioning, and the Clinician's General Impression), with a symptom severity rating that makes it possible to use the scale for periodic monitoring and for assessing long-term outcomes.

An evaluator absolutely blind to the type of protocol assessed the children prior to and after the treatment, and in the follow-up with intervals of one day, one week, four weeks and six months after first treatment.

The pre-treatment assessment of the first boy yielded a BSE score of 75 (with a score of 4 on item 18, regarding eating disorders), a CGI score for severity of 6 and a CARS score of 53. Then he received 6 intravenous injections, one each month, each injection containing 75 CU of porcine secretin in 7 ml. He was evaluated with the three rating scales at intervals of one day, one week, and four weeks from the beginning of treatment. In this case it was possible to assess treatment response after six months from first injection and a month after last injection.

The second case had a BSE score of 63 (with a score of 4 on item 18, regarding eating disorders), a CGI score for severity of 6 and a CARS score of 52 in his pre-treatment evaluation. He then received 2 intravenous injections with an interval of a month, each injection containing 50 CU of porcine secretin in 5 ml; then he was evaluated with the three rating scales at intervals of one day, one week, and four weeks from the beginning of treatment. Treatment was suspended after the second injection since no effect was reported. The follow-up at six months is not therefore comparable but was conducted in any case since the second injection had been administered.

## Results

In the first case, at interval 1 (after 1 day) there was no significant effect on the BSE, CGI (Severity was unchanged, the Global Improvement score was 4, i.e. no change, and the Efficacy Index score was 14, namely no treatment effect with no side effects), or CARS scores; at interval 2 (a week from first injection), we observed – clinically and through behavioral rating scales – a slight amelioration of his behavior, especially in alertness, expansion and efforts toward communication, while eye contact did not improve. In the same period parents reported an improvement in his diet, an associated problem; he began eating vegetables and pasta, which he had never wanted to eat before. He had a BSE score of 71, with a reduction from 4 to 3 on items 5, 6, 18 and 19. The CGI score for Severity did not change, the CGI score for Global Improvement dropped to 3, and the Efficacy Index score was 10 (mild effect with side effects that not interfere with the patient). His CARS score was 51.5 with a reduction of score at item 1, Relationships with People (4 to 3.5) at item 11, Verbal Communication (4 to 3.5), and at item 12, Nonverbal Communication (4 to 3.5). In the fourth week, at interval 3, these positive findings seemed to be still present; this was confirmed by a reduction of the BSE to 69, with a reduction on item 18 (eating disorders) from 3 to 1. We then proceeded with the second injection and since some slight amelioration of core symptoms was seen over the following four months we administered the other 4 planned intravenous injections. The symptoms follow-up after 6 months revealed enduring significant improvement with the same scores as at interval 3.

The second boy was evaluated prior to treatment and then at intervals of one day, one week, four weeks and six months after the treatment: no amelioration of core symptoms was noticed at any time of treatment in either eye contact, alertness or expansion of effort toward communication. Clinical observation confirmed the absence of positive results. We suspended treatment after the second injection in compliance with the parents' wishes.

At intervals 1 (one day from first injection), 2 (one week) and 3 (four weeks) there was no significant effect in the BSE, CGI (Severity was unchanged, the Global Improvement score was 4, i.e. no change and the Efficacy Index score was 14, i.e. no treatment effect with no side effects) or CARS (unvaried at 52) scores. Nor were any improvements reported after six months (Table 2).

**Table 2:** BSE score, CGI score for Severity, Global Improvement and Efficacy Index and CARS score at time 0, time 1 after one day, time 2 after one week, time 3 after four weeks and time 4 after six months.

Case	BSE score					CGI score S					CARS score				
	**0**	**1**	**2**	**3**	**4**	**0**	**1**	**2**	**3**	**4**	**0**	**1**	**2**	**3**	**4**

**1**	75	71	69	69	69	6	6	6	6	6	53	53	51.5	51.5	51.5
						**GI**	4	3	3	3					
						**EI**	14	10	10	10					
**2**	63	63	63	63	64	6	6	6	6	6	52	52	52	52	52
						**GI**	4	4	4	4					
						**EI**	14	14	14	14					

## Conclusion

The administration of secretin in our two young patients only led to some significant and enduring improvement on core symptoms in one case, where we observed significant changes in associated problems such as difficulties with toileting, sleeping and/or eating; laughing, crying or giggling at inappropriate times; response to touch, light, sound, taste or smells; unawareness of pain, heat or cold. In particular, we observed an evident amelioration of eating behavior associated with a stable global behavioral improvement.

This observation drew our attention to gastrointestinal symptoms and eating behavior, not only to dieting (even if in some cases it may be a relevant issue), in that the importance of social interaction and eating behavior has been pointed out by several studies [26]. Eating behavior in autistic children seems to be an intriguing subject for research, for several reasons:

a) because eating disorder, even when it is only considered as an associated problem, has important consequences on the health of these patients;

b) because of the important interaction between eating and social behavior; a recognized gastrointestinal disorder may contribute to the determination of behavioral problems in autistic patients.

Autistic disorder has always been considered to be substantially a neurobiological problem, but we cannot exclude different biological correlations, which could open up new perspectives in research and treatment of the disorder itself.

Besides, eating behavior is closely connected to attachment social behavior (i.e.: sucking is an act that includes the basis of eating and social behaviors, [27]). Studies conducted on animals [28] show that hormones such as oxytocin and vasopressin are involved in mediation at a central level of attachment behavior. A possible influence of secretin on autistic eating behavior could be mediated by the formation of unknown neuropeptides that, perhaps only in some children, finally have an influence on the construction of social behavior.

On the other hand Horvath *et al*. [29] found gastroesophageal reflux and reflux esophagitis to be the most frequently detected gastrointestinal abnormalities in children with autistic disorder (69.4%). In our report the child who improved with secretin treatment was the one characterized by reflux esophagitis, while the child who did not improve had chronic diarrhea but no gastric or esophageal reflux. It is known that secretin has a suppressive effect on gastric secretion [30]. Whether a low level of secretin may contribute to the high prevalence of acid reflux needs further investigations, but our report confirms the suggestion of Horvath & Perman [31] that gastrointestinal abnormalities may contribute to some of the behavioral problems, and the presence of esophagitis correlates well with the reported symptoms and may in part explain the sudden irritability, crying behavior and diet restriction as shown in our first case. In some way the behavioral problems connected to gastrointestinal abnormalities could represent a form of challenging behavior where a simple correction of dieting, or reflux reduction could determine great behavioral improvements [32]. It is also possible that autistic children with gastroesophageal reflux and esophagitis and higher IQ constitute a subtype, and probably respond better to secretin administration. Following this line of investigation, recent studies on autism suggest that there may be different subtypes of autism, with special reference to the "gastrointestinal subtype" [19].

We are justified in thinking that a more precise definition of each single case of autistic disorder would contribute to delineating possible subtypes of autistic disorder, which is currently a rather non-specific and over-inclusive diagnostic category. It is also true that many studies [33] show an increasing prevalence of PDD, especially among people with intellectual disability; this could in part be due to a better application of PDD DSM-IV criteria for autistic disorder or PDD not otherwise specified, and in part to increasing interest in the concept of the autistic spectrum. As a result of the relative non-specificity of the autistic disorder category, studies conducted on large samples may have the bias of studying non-homogenous subjects. Consequently, carefully described case reports could provide orientation regarding possible subtypes, so these can be described and recognized, and specific diagnostic criteria developed. The higher IQ index, and the presence or absence of some gastrointestinal symptoms in this case could represent indications of a somewhat specific subtype of autistic disorder, susceptible to specific treatments.

To conclude, secretin is not an effective cure for autism, as the extensive media attention suggested at the very beginning. However, observation of its efficacy on specific targets permits a number of reflections: it is only by using anecdotal reports and case observations showing reduction in the severity of specific symptom domains within autistic disorders, recognizing different specific subtype symptom domains, in order to elaborate good and valid study design with accurate selection of patients and specific outcome measures that it will be possible to obtain progress in the comprehension and cure of autism [34].
